# Describing a new food group classification system for UK biobank: analysis of food groups and sources of macro- and micronutrients in 208,200 participants

**DOI:** 10.1007/s00394-021-02535-x

**Published:** 2021-03-25

**Authors:** Carmen Piernas, Aurora Perez-Cornago, Min Gao, Heather Young, Zoe Pollard, Angela Mulligan, Marleen Lentjes, Jennifer Carter, Kathryn Bradbury, Tim J. Key, Susan A. Jebb

**Affiliations:** 1 Nuffield Department of Primary Care Health Sciences, University of Oxford, Radcliffe Primary Care Building, Radcliffe Observatory Quarter, Woodstock Road, Oxford, OX2 6GG UK; 2 Cancer Epidemiology Unit, Department of Population Health, University of Oxford, Nuffield, UK; 3 School of Public Health, Peking University, Beijing, China; 4 Department of Public Health & Primary Care, Institute of Public Health, University of Cambridge, Cambridge, UK; 5 NIHR BRC Diet, Anthropometry and Physical Activity Group, MRC Epidemiology Unit, University of Cambridge, Cambridge, UK; 6 School of Medical Sciences, Clinical Epidemiology & Biostatistics, Örebro University, Örebro, Sweden; 7 Nuffield Department of Population Health, University of Oxford, Oxford, UK; 8 School of Population Health, National Institute for Health Innovation, University of Auckland, Auckland, New Zealand

**Keywords:** Food groups, Dietary intake, Macronutrients, Micronutrients

## Abstract

**Purpose:**

The UK Biobank study collected detailed dietary data using a web-based self-administered 24 h assessment tool, the Oxford WebQ. We aimed to describe a comprehensive food grouping system for this questionnaire and to report dietary intakes and key sources of selected nutrients by sex and education.

**Methods:**

Participants with at least one valid 24-h questionnaire were included (*n* = 208,200). Dietary data were grouped based on the presence of nutrients as well as culinary use, processing, and plant/animal origin. For each food group, we calculated the contribution to energy intake, key macronutrients, and micronutrients. We also identified the top contributors to energy intake, free sugars and saturated fat by sex and education.

**Results:**

From the 93 food groups, the top five contributors to energy intake (in descending order) were: desserts/cakes/pastries; white bread; white pasta/rice; bananas/other fruit; semi-skimmed milk. Wine, beer, and fruit juices were the top beverage contributors to overall energy intake. Biscuits, and desserts/cakes/pastries were the highest contributors to free sugars, total fat, and saturated fat intakes, but also contributed to the calcium and iron intakes. Top contributors to energy, saturated fat, and free sugars were broadly similar by sex and education category, with small differences in average nutrient intakes across the population.

**Conclusion:**

This new food classification system will support the growing interest in the associations between food groups and health outcomes and the development of food-based dietary guidelines. Food group variables will be available to all users of the UK Biobank WebQ questionnaire.

**Supplementary Information:**

The online version contains supplementary material available at 10.1007/s00394-021-02535-x.

## Introduction

A poor diet is one of the most important modifiable risk factors for chronic disease, especially cardiovascular disease, diabetes, and cancer [1, 2]. Most of the existing evidence relates to associations between individual nutrients or foods and health outcomes, and hence dietary guidelines have traditionally been based on nutrient recommendations. However, people consume multiple foods and nutrients that may interact with each other, and studying overall dietary patterns may be more relevant to understand health risk [3]. Accordingly, there is growing interest in developing food-based dietary guidelines, which may also be easier to communicate to consumers to support changes towards more healthful dietary patterns.

Dietary habits are shaped by individual preferences, social context and cultural norms and may reveal differences not apparent in nutrient intakes. A previous study using purchasing data from UK households showed that the overall saturated fat content of purchases did not differ between socio-economic groups, however, there was a higher proportion of energy from cheese and dairy among higher socioeconomic status (SES) households, but a higher proportion of energy from sweet snacks and puddings among lower SES households [4]. These differences in food patterns may be associated with different health outcomes since dairy products will also contribute important micronutrients such as calcium, while sweet snacks will also be high in free sugars. Studying the selection of foods and drinks also provides insights into eating behaviours which might offer new approaches to interventions to support a healthier diet.

The UK Biobank study collected detailed measures of dietary intake using a web-based self-administered 24 h dietary assessment tool, the Oxford WebQ [5, 6]. Although this is a unique resource for the study of diet and disease risk, this dietary questionnaire is not easy to work with because it does not have a comprehensive food grouping system. The aims of this study were: 1) to describe the development of a food grouping system to classify the foods consumed by the UK Biobank participants; 2) to describe the major food sources of energy, macro- and main micronutrients in the whole population, as well as by sex and educational attainment.

## Methods

### Study Population

The UK Biobank study is a national prospective cohort in the UK involving 502,655 participants aged 40–69 years at baseline who were recruited between 2006 and 2010 [7]. Approximately 9.2 million eligible adults living within 25 miles of the UK Biobank assessment centres (England, Wales and Scotland) were invited by letter through NHS central registries [8]. Participants who volunteered to take part (5.5% response rate) completed a full baseline assessment with self-reported measurements via touch-screen questionnaires as well as a verbal interview. A wide range of information on socio-demographic factors, lifestyle, and behavioural factors including a short food-frequency questionnaire and medical history were collected, along with physical measurements (such as height and weight), blood and urine samples.

UK Biobank protocols and study details can be found elsewhere (http://www.ukbiobank.ac.uk/wp-content/uploads/2011/11/UK-Biobank-Protocol.pdf) [7]. The UK Biobank study was conducted according to the Declaration of Helsinki and ethical approval was granted by the North West Multi-Centre Research Ethics Committee (reference number 06/MRE08/65). At recruitment, all participants gave informed consent to participate and be followed-up through data-linkage.

### Dietary assessment – Oxford WebQ

Towards the end of the baseline assessment period (April 2009-September 2010), the UK Biobank started collecting detailed dietary intake measures using a web-based self-administered 24 h dietary assessment, the Oxford WebQ. The Oxford WebQ was completed by 70,724 participants attending their baseline assessment. Additionally, between 2011 and 2012 all participants with valid email addresses (*n* = 331,013) were invited to complete the Oxford WebQ on four separate occasions. This was done every 3–4 months on variable days to maximise the coverage by season and day of the week (cycle 1: February 2011 to April 2011; cycle 2: June 2011 to September 2011; cycle 3: October 2011 to December 2011; cycle 4: April 2012 to June 2012). Approximately 53% of the participants (*n* = 176,012) who were contacted by email completed at least one assessment, with a total of 211,050 participants completing at least one dietary assessment either online or at the baseline assessment [6].

The Oxford WebQ collects information on foods and beverages consumed over the previous day. Participants were presented with a list of up to 206 foods and 32 beverages commonly consumed in the UK and selected the number of portions consumed from each food. This food list was constructed using information from the UK National Diet and Nutrition Survey (NDNS) as well as a pilot study [5]. At the start of the questionnaire, participants indicated whether their diet over the previous day was typical and if they were following a special diet. Descriptions and help sections were used to help estimating portion sizes of foods (e.g. slices, cups, servings) and participants were asked to report ingredients of composite dishes separately. Total energy and nutrient intakes were generated by multiplying the number of portions consumed by the set quantity of each food portion size and its nutrient composition obtained from the UK Nutrient Databank Food Composition Tables (FCT) from survey year 6, (2012–2013 and 2013–2014) [9–11]. Dietary fibre was calculated using the Englyst method [12], which includes non-starch polysaccharides but not lignin and resistant starches.

### Food group system

We classified the 206 foods and 32 beverages reported in the Oxford WebQ into 93 groups (79 food and 14 beverage groups) belonging to 15 main food categories (13 food and 2 beverage categories, Supplemental Table S1). This food group system was mainly based on the classification used in the UK NDNS but many food groups were further disaggregated to offer the potential to investigate a variety of specific research questions which are related to nutrients which may be differently related to health when consumed as part of different foods; such as free sugars, saturated fat or fibre, as well as culinary use; and the extent of processing or plant/animal origin. For cereal and cereal products, we separated breads and pasta by fibre content (e.g. white and wholemeal), while breakfast cereals were divided by type of cereal (e.g. oat and wheat based) and sugar content. Mixed dishes were divided into pizza, other cereal-based dishes with added fat, Indian snacks, sushi and soups, recognising the differences in fat content of each dish as well as their different cultural and culinary roles. For dairy and dairy products, we separated milk, cheese and yogurt by fat content (e.g. higher and medium/lower fat). Fats, butter and spreads were separated by fat content but also by the source of fat (e.g. animal and vegetable fat). Meat and meat products were divided by the type of animal (e.g. beef and pork) except for the group processed meat which may include more than one animal source. Fish and fish dishes were separated into white fish/tinned tuna, oily fish and battered fish. Meat substitutes were divided into soy-based and other vegetarian meals. Vegetables were separated into groups which considered micronutrient, carbohydrate or protein content (e.g. green leafy/cabbage, root (excluding potatoes), tomatoes, allium, legumes) as well as fat content (e.g. baked/boiled potatoes; mashed potatoes and fried/roast potatoes). Fruits were grouped according to micronutrient content (e.g. citrus; berries and apples/pears), as well as processing (e.g. dried and stewed fruit). Nuts and seeds were divided according to the salt content. Sugary foods were divided into groups reflecting differences in consumption: added sugars/preserves (including table sugar); chocolate confectionery; other sweets (non-chocolate); biscuits/cookies; desserts, cakes and pastries; milk-based desserts; soy-based desserts; and sweet spreads (including chocolate and peanut-butter spreads). Sauces and condiments were divided by the fat content. Beverages were first separated into alcoholic and non-alcoholic. Non-alcoholic beverages were further separated by the caffeine content, sugar content, or presence of milk. Where possible, milk added to coffee/tea and porridge was disaggregated to be included in the milk group. For other milk-based drinks this was not possible and they were categorised in their own group. Alcoholic drinks were then divided into wine, beer/cider, and spirits.

The final food group classification was further refined and consolidated following consultation with three nutrition scientists from the UK with experience in nutritional epidemiology or public health policy.

### Exclusions

Participants who completed a minimum of one valid WebQs were included. Participants with implausible energy intakes (over- and under-reporters) were excluded before analysis. We used the individualised method to calculate the ratio of reported energy intake (EI) to estimated energy requirement (EER) (EI:EER), where EERs were calculated using the Schofield Equation [13]. 95% CIs were calculated to classify individuals as plausible reporters (EI:EER within the 95% CIs), over-reporters (EI:EER > upper 95% CI) or under-reporters (EI:EER < lower 95% CI).

### Demographic and lifestyle characteristics

Demographic and lifestyle characteristics were collected at baseline using a touchscreen questionnaire and were categorised as: White ethnicity vs other (including Asian, Black, mixed background); education was classified as higher degree (college or university degree, or professional qualifications) vs any school degree (A levels, AS levels, O levels, GCSEs or CSEs) vs vocational qualifications (NVQ, HND or HNC), vs no qualifications or other not classified elsewhere; physical activity was categorised as high (≥ 3000 metabolic equivalent (MET)-minutes per week) vs moderate (≥ 600 and < 3000 metabolic equivalent (MET)-minutes per week) vs low (< 600 metabolic equivalent (MET)-minutes per week); smoking was categorised as never, current, previous; alcohol intake was categorised as 5 + units/week, 1–4 units/week, < 1 units /week, never. Body mass index (BMI) was calculated using the measured height and weight and categorised as: underweight [< 18.5 kg/m^2^), healthy weight [18.5 to < 25 kg/m^2^], overweight [25 to < 30 kg/m^2^], obesity [> 30 kg/m^2^].

### Statistical analyses

We calculated the individual’s mean intakes from all their completed dietary assessments. Descriptive statistics (crude means, SD) were computed for total daily energy (kJ/day), weight or volume of food and beverages (g or ml/day), as well as for macronutrients (g/day) and micronutrients (mg or µg/day). For each food and beverage group, we calculated per capita intakes as well as mean intakes among consumers only, including only the participants consuming more than 0 g or kJ from each food group. To identify the major sources of energy intake, the per capita contribution of each food or beverage group to total daily intake was calculated as the energy consumed from each food group divided by the total daily energy. We finally investigated the top food group sources of energy, saturated fat, and free sugars by sex and education status. Stata version 14 (StataCorp LP) was used for all analysis.

## Results

A final sample of 208,200 participants, 56.1 (SD 7.9) years old at recruitment, was included in the analysis after the exclusion of participants who did not provide any dietary data (*n* = 292,136) and under- or over-reporters (*n = *2,319). The majority of the study population was white (95%), 48% reported a higher education degree, 36% reported high levels of physical activity, 8% were current smokers, and 23% drank 5 + alcohol units/week. The mean body mass index was 27 (SD 5) kg/m^2^ at recruitment, with 42% classified as overweight and 21% as obese (Supplemental Table S2).

### Top sources of energy and macro- and micronutrients

The ten major contributors to EI per capita (in descending order) were (Table 1): desserts, cakes, and pastries (5.2% EI); white bread (3.7% EI); white pasta & rice (3.1% EI); bananas & other fruits (other than citrus, berries, apples and pears, 3.1%EI); semi-skimmed milk (3% EI); biscuits (2.9% EI), fried/roast potatoes (2.9% EI); wholemeal bread (2.7% EI) and mixed bread (2.7% EI); and high-fat cheese (2.7% EI). Among the beverage subcategories, the top contributors to energy intake were fruit juice (1.8% EI) among non-alcoholic beverages and red wine (2.4% EI) and beer/cider (2.4% EI) among alcoholic beverages. However, water/sparkling water (513 g/d) and tea (425 g/d) were the top contributors to beverage volume per day. Overall, the categories of cereal and cereal products, followed by the sugar, preserves, cakes & confectionery and vegetables and potatoes were the top 3 categories contributing the most to the total energy intakes (Table 1).

**Table 1:** Food group consumption (per capita and per consumer) among all UK Biobank participants

	Per capita *n *= 208,200			Per consumer		
	Mean intake g/d (SD)	Mean intake kJ/d (SD)	% Energy intake	%	Mean intake g/d (SD)	Mean intake kJ/d (SD)
Total daily intake	3229 (794)	8679 (2422)	100	100	3229 (794)	8679 (2422)
1-Cereals and cereal products	206.2 (115.0)	1817.1 (882.8)	20.9	99	209.2 (113.1)	1843.4 (861.4)
White bread	30.2 (47.8)	319.7 (507.3)	3.7	46	65.9 (51.3)	698.0 (545.7)
Wholemeal bread	25.0 (41.1)	238.1 (392)	2.7	42	60.1 (44.1)	571.7 (422.1)
Mixed bread, brown and seeded	23.5 (38.3)	232.3 (379.8)	2.7	42	56.0 (41.1)	553.8 (407.2)
Other bread	9.2 (27.6)	106.3 (314.0)	1.2	20	45.6 (45.9)	527.0 (516.9)
Savoury crackers	4.7 (10.6)	80.4 (181.4)	0.9	26	18.2 (13.6)	308.7 (236.5)
Bran cereal	4.0 (11.6)	55.6 (160.6)	0.6	14	28.5 (16.0)	394.8 (222.0)
Biscuit cereal	5.5 (13.6)	82.1 (203.4)	0.9	19	29.2 (17.1)	436.1 (255.6)
Oat cereal (non-sugar)	34.2 (69.5)	119.1 (250.0)	1.4	24	142.3 (68.6)	494.7 (271.7)
Oat cereal (sugar)	2.4 (10.8)	40.3 (182.0)	0.5	7	36.6 (23)	617.8 (388.5)
Muesli	10.5 (22.9)	151.8 (332.1)	1.7	22	47.0 (25.2)	682.7 (365.4)
Other cereal (sugar)	4.8 (11.1)	76.5 (174.2)	0.9	22	22.5 (13.2)	355.7 (204.4)
White pasta & rice	43.6 (70.0)	269.0 (436.9)	3.1	39	111.1 (70.7)	685.2 (448.4)
Wholemeal pasta, brown rice and other wholegrains	8.5 (33.2)	46.0 (181.7)	0.5	9	91.3 (65.4)	494.7 (365.3)
2-Mixed-dishes	58.0 (93.4)	369.8 (716.0)	4.3	51	113.3 (103.7)	722.0 (864.0)
Pizza	10.8 (52.1)	123.8 (598.4)	1.4	7	151.7 (129.8)	1742 (1489.6)
Grain dishes—added fat	7.6 (18.8)	143.1 (338.4)	1.6	26	29.0 (26.9)	546.4 (465.6)
Samosa, pakora	0.9 (6.8)	10.0 (79.2)	0.1	3	31.2 (27.1)	361.8 (314.4)
Soups	37.6 (74.5)	85.0 (185.9)	1.0	28	133.9 (82.8)	302.3 (239.3)
Sushi	1.2 (17.4)	7.8 (118.3)	0.1	1	164.3 (128.0)	1117.5 (870.5)
3-Dairy and dairy-free products	271.1 (145.3)	795.1 (453.7)	9.2	98	275.9 (142.0)	809.3 (445.0)
Whole milk	13.7 (60.8)	37.0 (165.0)	0.4	8	181.7 (136.5)	492 (372.1)
Semiskimmed milk	140.9 (139.9)	262.9 (260.2)	3.0	67	209.9 (121.2)	391.5 (224.7)
Skimmed milk	46.2 (102.8)	66.9 (147.5)	0.8	24	195.1 (124.7)	282.4 (176.2)
Rice/oat drink	0.8 (11.9)	1.2 (19.0)	0.0	1	109.3 (90.5)	175.9 (142.9)
Soy drink	7.5 (40.1)	13.8 (74.7)	0.2	5	149.1 (104.8)	275.6 (197.5)
Full fat yogurt	9.6 (27.5)	38.9 (111.0)	0.4	17	56.3 (42.3)	227.5 (170.9)
Low fat yogurt	33.9 (53.9)	99.2 (158.1)	1.1	40	85.4 (54.2)	250.4 (158.8)
High fat cheese	14.4 (19.0)	231.2 (307.1)	2.7	55	26.1 (18.7)	419.9 (303.4)
Medium and low fat cheese	2.9 (9.9)	25.5 (87.8)	0.3	15	20.0 (18.2)	175.6 (163.4)
Cream	1.2 (4.6)	18.5 (71.1)	0.2	8	14.5 (8.1)	223 (123.8)
4-Egg and egg dishes	21.5 (40.4)	163.7 (316.1)	1.9	37	57.7 (48.0)	440.4 (383.3)
5-Fat and spreads	11.9 (10.7)	269.2 (274.9)	3.1	78	15.3 (9.8)	344.6 (266.0)
Olive oil (drizzling/dunking)	0.3 (1.3)	10.9 (49.8)	0.1	6	5.0 (2.7)	183.4 (101.3)
Dairy fat spread lower fat	0.9 (3.9)	14.2 (62.9)	0.2	9	10.5 (8.9)	165.5 (144.7)
Dairy fat spread	5.2 (9.3)	150.0 (272.2)	1.7	37	13.9 (10.5)	403.6 (311.4)
Vegetable spread lower fat	2.4 (5.8)	33.4 (80.4)	0.4	23	10.2 (7.9)	142.4 (109.8)
Vegetable spread	3.1 (6.5)	60.6 (126.4)	0.7	30	10.3 (8.0)	199.4 (157.5)
6-Meat and meat products	92.6 (72.0)	805.0 (656.8)	9.3	82	112.7 (63.5)	979.6 (594.9)
Poultry	30.9 (50.1)	218.1 (356.4)	2.5	40	78.1 (51.5)	550.2 (371.1)
Pork	8.5 (26.8)	73.2 (234.1)	0.8	14	61.6 (44.1)	530.2 (393.6)
Beef	22.9 (41.5)	207.7 (379.7)	2.4	33	68.9 (44.8)	624.5 (416.2)
Lamb	6.7 (24.5)	74.0 (271.5)	0.9	11	61.9 (45.5)	679.4 (515.3)
Other meat, offal	1.8 (11.2)	25.7 (156.6)	0.3	4	44.2 (33.9)	616.1 (472.7)
Processed meat	18.0 (30.0)	171.7 (313.4)	2.0	47	38.6 (33.7)	367.4 (371.8)
Breaded/battered chicken	3.7 (18.6)	34.7 (175.1)	0.4	7	56.3 (48.1)	530.7 (453.4)
7-Fish and fish dishes	32.4 (48.0)	225.6 (358.1)	2.6	4	69.5 (48.7)	484.3 (387.2)
White fish and tinned tuna	11.9 (28.8)	63.1 (156.5)	0.7	22	54.5 (38.3)	289.7 (216.1)
Shellfish	3.2 (12.7)	10.4 (42.3)	0.1	11	29.0 (26.7)	93.9 (91.3)
Oily fish	11.3 (27.4)	97.5 (236.3)	1.1	21	53.3 (36.2)	458.7 (311.6)
Breaded/battered fish	5.9 (24.8)	54.5 (227.1)	0.6	9	68.9 (52.6)	632.1 (482.4)
8-Meat substitutes	4.1 (20.5)	33.4 (175.0)	0.4	6	64.5 (51.9)	523.0 (473.2)
Vegetarian meals	3.5 (19.0)	31.2 (171.9)	0.4	5	65.1 (51.9)	578.9 (480.1)
Soy-based meals	0.6 (6.4)	2.1 (22.5)	0.0	1	42.8 (32.8)	151.4 (116.3)
9-Vegetables and potatoes	306.0 (207.1)	906.8 (691.0)	10.4	92	331.4 (195.0)	982.0 (665.8)
Raw salad	20.6 (32.1)	11.5 (18.1)	0.1	50	41.3 (34.9)	23.1 (19.7)
Green leafy/cabbages	29.2 (47.7)	31.6 (53.1)	0.4	47	62.7 (52.9)	67.9 (59.9)
Root vegetables	21.1 (30.7)	26.4 (45.6)	0.3	54	39.3 (32.3)	49.1 (52.5)
Tomatoes	38.6 (54.2)	34.7 (51.3)	0.4	56	69.6 (56.0)	62.4 (54.8)
Allium vegetables	11.8 (20.8)	43.9 (75.6)	0.5	46	25.3 (24.3)	94.5 (86.8)
Other vegetables (mushrooms, fruiting, mixed)	59.1 (69.8)	115.9 (170.3)	1.3	72	81.6 (70.0)	160.0 (181.6)
Peas/sweetcorn	8.6 (17.8)	24.4 (51.1)	0.3	30	28.5 (22.1)	81.3 (63.8)
Potatoes/sweet potatoes (baked/boiled)	48.8 (75.8)	187.0 (309.8)	2.2	45	109.2 (79.2)	418.7 (343.3)
Mashed potatoes	17.4 (44.1)	74.6 (191.4)	0.9	20	88.5 (60.4)	380.3 (265.4)
Fried/roast potatoes	31.3 (61.4)	250.0 (491.2)	2.9	32	97.4 (72.9)	778.9 (582.8)
Legumes/pulses	13.7 (31.0)	49.5 (109.6)	0.6	28	48.1 (41.5)	174.4 (143.2)
Vegetable side dishes	4.7 (18.2)	42.7 (164.5)	0.5	11	42.8 (37.1)	386.3 (335.1)
Vegetable dips	1.2 (4.5)	14.6 (55.9)	0.2	8	14.1 (7.9)	173.4 (98.1)
10-Fruits	215.5 (170.5)	528.8 (425.4)	6.1	89	242.7 (161.7)	595.5 (405.0)
Citrus	32.2 (54.2)	51.0 (85.3)	0.6	39	82.6 (58.2)	131.0 (90.6)
Berries	8.4 (18)	11.5 (25.0)	0.1	30	27.7 (23)	37.7 (32.7)
Apples and pears	61.0 (79.5)	107.6 (138.1)	1.2	54	113.6 (76)	200.4 (130.0)
Bananas and other fruit	94.1 (97.2)	265.2 (257.6)	3.1	74	127.7 (92.4)	359.9 (236.5)
Dried fruit	7.3 (20.1)	67.2 (183)	0.8	21	34.8 (31.2)	320.6 (280.2)
Stewed fruit	12.5 (33.5)	26.3 (72.6)	0.3	19	64.0 (49.5)	135.2 (111.2)
11-Nuts and seeds	6.9 (15.2)	172.0 (384.3)	2.0	41	16.7 (20.0)	417.9 (506.1)
Salted nuts and seeds	2.6 (10.3)	66.7 (263.1)	0.8	11	23.7 (21.7)	605.5 (549.3)
Unsalted nuts and seeds	4.2 (11.0)	105.2 (276.3)	1.2	35	12.1 (15.9)	299.1 (398.8)
12-Sugar, preserves, cakes and confectionery, snacks	100.0 (79.9)	1468.5 (1121.6)	16.9	94	106.5 (78.1)	1564.5 (1090.9)
Added sugars and preserves	9.1 (14.0)	130.4 (219.0)	1.5	54	16.9 (15.2)	241.9 (249.0)
Chocolate confectionery	9.6 (19.9)	190.3 (393.7)	2.2	40	24.2 (25.4)	481.3 (501.9)
Other sweets	2.7 (12.2)	42.3 (193.3)	0.5	13	20.3 (27.6)	318.9 (439.7)
Savoury snacks	9.4 (16.8)	193.4 (346.6)	2.2	36	26.0 (18.7)	536.3 (386.4)
Biscuits	12.5 (18.7)	250.8 (376.4)	2.9	48	25.9 (19.5)	520.4 (392.0)
Milk-dairy desserts	23.9 (46.3)	197.0 (378.9)	2.3	32	73.6 (54.2)	606.7 (439.8)
Desserts and cakes and pastries	31.7 (41.0)	451.6 (598.1)	5.2	59	53.7 (40.8)	764.1 (605.4)
Soya-based desserts and yogurt	0.7 (8.3)	3.6 (42.1)	0.0	1	64.4 (46.9)	327.3 (238.3)
Nut-based spreads	0.4 (2.0)	9.1 (48.2)	0.1	4	8.6 (4.6)	205.8 (109.7)
13-Sauces and condiments	22.1 (27.7)	149.2 (192.9)	1.7	62	35.6 (27.4)	240.4 (195.0)
Sauces (higher fat)	13.1 (18.5)	110.4 (169.1)	1.3	49	26.5 (18.4)	223.8 (180.5)
Sauces (lower fat)	9.0 (19.2)	38.8 (75.3)	0.4	36	25.0 (24.9)	107.1 (91.3)
14-Non-alcoholic beverages	1617.6 (521.7)	372.9 (416.2)	4.3	90	1617.9 (521.3)	412.7 (418.7)
Fruit juice	105.9 (144.3)	159.7 (217.7)	1.8	52	204.2 (141.8)	308 (213.8)
Coffee, caffeinated	240.9 (268)	6.5 (9.5)	0.1	48	354.2 (255.8)	13.4 (9.7)
Coffee, decaffeinated	55.4 (153.2)	1.7 (5.2)	0.0	15	297.3 (232.7)	11.1 (8.6)
Tea	425.4 (349.7)	2.6 (14.2)	0.0	6	557.1 (294.5)	44.1 (40.4)
Tea, decaffeinated	78.9 (187.1)	0.0 (0.0)	0.0	24	323.2 (253.8)	0.0 (0.0)
SSBs and other sugary drinks	90.7 (182.8)	120.3 (246.1)	1.4	36	254.8 (228.2)	338.2 (310.7)
Low/non sugar SSBs	72.0 (197.8)	1.3 (3.5)	0.0	21	348.8 (304.9)	6.1 (5.3)
Water/sparkling water	513.7 (396.4)	0.0 (0.0)	0.0	89	579.4 (373.1)	0.0 (0.0)
Milk-based and powdered drinks	34.9 (96.7)	80.8 (224.6)	0.9	22	161.8 (151)	374.4 (351.7)
15-Alcoholic beverages	263.1 (433.6)	602.2 (795.5)	6.9	63	417.9 (483.7)	956.5 (816.2)
White wine	43.5 (109.6)	150.0 (377.9)	1.7	23	186 (157.7)	641.3 (543.7)
Red wine	64.5 (129.3)	204.6 (410.1)	2.4	33	196.7 (158.2)	623.6 (501.3)
Fortified wine	1.0 (7.0)	5.4 (38.3)	0.1	4	27.4 (25.2)	149.5 (137.7)
Beer and Cider	150.6 (400.9)	207.2 (551.8)	2.4	25	609.8 (608.9)	839.3 (838.1)
Spirits	3.5 (12.0)	34.9 (119.6)	0.4	15	23.4 (22.3)	232.8 (222.0)

Units for beverages are ml/day

**Fig. 1:**
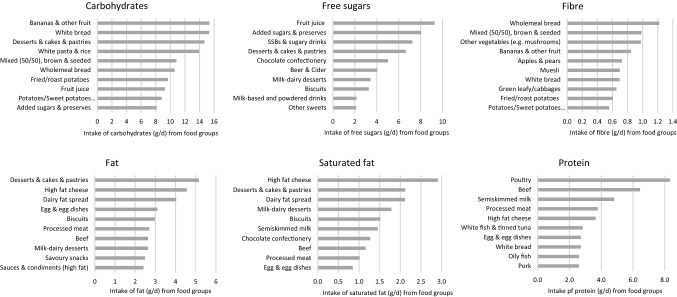
Top ten food contributors to total carbohydrates, free sugars, fibre, total fat, saturated fat and total protein per capita among all UK Biobank participants

Figure 1 shows the top food contributors to relevant macronutrients. Desserts, cakes and pastries were top contributors to total carbohydrate, free sugar, total fat and saturated fat intakes (Fig. 1, Supplemental Table S3). The bread was the top contributor to total carbohydrate (white, mixed) while wholemeal and mixed bread was the top contributor to fibre intake. Bananas & other fruits were major contributors to both total carbohydrates and fibre intakes. High fat cheese was a top contributor to total fat, saturated fat, and protein intake. Dairy fat spreads (e.g. butter) were top contributors to both total fat and saturated fat. Poultry and beef were the top sources of total protein intakes. Semi-skimmed milk was an important contributor to many micronutrients, including vitamin B12, calcium and potassium intakes (Supplemental Table S4). Overall, fruits and vegetables, as well as fruit juice made a substantial contribution to intakes of folate, vitamin C, and potassium. Beef, oily fish were also important contributors to vitamin B12, and beef alongside bread and cereals were important contributors to intakes of iron.

### Sources of energy, saturated fat and free sugars by sex and education status

**Fig. 2:**
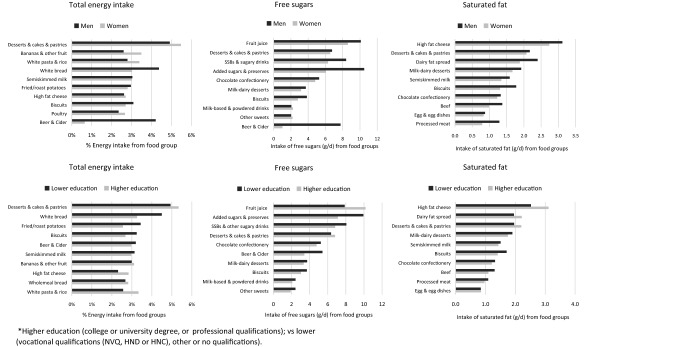
Top ten food group contributing to total energy intake per capita (%), saturated fat and free sugars (g/d) by sex and education

Total daily energy and alcohol intake were higher in men than in women, but the proportion of energy from macronutrients was similar (Table 2). The top ten foods contributing the most to energy intake were similar in men and women (Fig. 2), with desserts, cakes and pastries ranking at the top. Among men, white bread and beer/cider ranked 2nd and 3rd; whereas in women bananas and other fruit and white pasta/rice ranked 2nd and 3rd. Fruit juice was the top contributor to free sugars in women, while added sugars/preserves was the top contributor among men. For SFA intake, high fat cheese, desserts and cakes, and dairy-fat spreads provided the most SFA to the diets among men and women.

**Table 2:** Average energy, macronutrient and micronutrient intakes by sex and education status among UK Biobank participants

	Total	Women	Men	Lower education	Higher education
	*n* = 208,200	*n* = 114,965	*n* = 93,235	*n* = 106,874	*n* = 100,336
Energy intake (kJ/day), mean (SD)	8679 (2422)	8046 (2103)	9460 (2558)	8652 (2494)	8713 (2339)
Volume intake (g/day), mean (SD)	3229 (794)	3142 (721)	3336 (863)	3202 (820)	3259 (763)
Protein (% energy), mean (SD)	15.9 (3.5)	16.2 (3.6)	15.5 (3.4)	15.9 (3.6)	15.8 (3.4)
Total fat (% energy), mean (SD)	31.5 (6.7)	31.8 (6.7)	31.1 (6.6)	31.4 (6.7)	31.6 (6.5)
Saturated fat (% energy), mean (SD)	11.6 (3.3)	11.7 (3.3)	11.6 (3.3)	11.6 (3.4)	11.7 (3.3)
Carbohydrates (% energy), mean (SD)	49.5 (8.3)	49.8 (8.3)	49.0 (8.3)	49.6 (8.3)	49.3 (8.2)
Free sugars (% energy), mean (SD)	11.6 (5.7)	11.0 (5.5)	12.3 (5.8)	11.8 (5.9)	11.3 (5.3)
Englyst fibre g/day, mean (SD)	17.9 (6.6)	17.6 (6.4)	18.3 (6.9)	17.5 (6.7)	18.3 (6.5)
Alcohol g/day, mean (SD)	17.1 (22.0)	12.9 (17.3)	22.3 (25.7)	16.9 (22.7)	17.3 (21.2)

Higher education (college or university degree, or professional qualifications); vs. lower (vocational qualifications (NVQ, HND or HNC) or no qualifications)

Total daily energy and macronutrient intakes were similar regardless of education (Table 2). Desserts, cakes and pastries and white bread were the top sources of energy intake in both education groups (Fig. 2). A few small differences were noted. Among people with higher education, white pasta/rice and higher fat cheese provided a higher proportion of energy intake, while those with lower education obtained a higher proportion of energy from biscuits, white bread, fried/roast potatoes and beer/cider. Fruit juice contributed more free sugars among people with higher education, whereas added sugars and preserves, sugars-sweetened beverages (SSB) and beer & cider provided more free sugars among those with lower education. Differences in SFA sources between education groups were usually small (< 0.6 g), but high fat cheese, dairy fats, desserts & cakes made a greater contribution to saturated fat in the diets of people with higher education, whereas biscuits, chocolate confectionery and beef provided slightly more saturated fat to the diet among those with lower education.

## Discussion

We have developed a comprehensive food grouping system to help analyse the UK Biobank dietary data. We created 93 distinctive food groups belonging to 15 main food categories (13 food and 2 beverage categories). The proposed food groups were designed to broadly align with the UK NDNS food group system, but have been expanded to meet the needs of researchers with interests in diverse health outcomes (e.g. different types of red meat, animal vs plant sources, saturated fat, free sugars or fibre content). The top contributors to energy, saturated fat and free sugars were broadly similar by sex and level of education, reflecting small differences in nutrient intakes.

Detailed measures of dietary data were collected for a large sample of the UK Biobank population using the Oxford WebQ which is a major strength. The development of a comprehensive food grouping system is rather complex due to the wide availability of products that people consume, and the wide diversity in food preparation and consumption habits. In addition, foods contain multiple sources and varying levels of important macro and micro-nutrients with differential effects on health which were considered in this study. Although the Oxford WebQ was based on a fixed list of foods, it captures sufficient detail to separate foods by free sugar content, saturated fat or fibre, as well as by culinary use, processing or plant/animal origin, but these groupings could also be combined for specific analyses.

An important limitation of using a fixed list of foods and beverages in the Oxford WebQ is that it can increase the likelihood of missing foods which are not on the list, although estimates of total energy intakes reported here were not notably low suggesting that it still captures dietary intake reliably as shown by comparison with recovery biomarkers and interviewer-administered 24 h recalls [6, 14]. However, there are other sources of measurement error related to self-reporting of dietary intake. Firstly, each WebQ collected dietary intake over the previous 24 h which is not representative of usual intake. Here we included all participants who provided at least one dietary assessment, which will be affected by random error related to day-to-day variability, although this is usually not problematic when calculating population averages [15] which was the main aim of this study. However, it is important to use several 24-h dietary assessments when assessing diet-disease associations in future studies in this cohort in order to capture usual intakes. Systematic error related to over- and/or underreporting of dietary intake will likely affect our estimates. This bias can be introduced for example when participants forget to report their dietary intake (although this is less problematic when reporting diet over the previous 24 h), or deliberately under or over-report specific foods and beverages [15]. Regarding the representativeness of the sample, previous analyses have suggested that the participants completing more dietary assessments tended to be older and more educated compared to the general population of the UK Biobank, and this may have limited the ability to detect differences across education groups [6]. However, the direction of risk factor associations in the UK Biobank seem to be generalizable to the wider UK population [16].

In interpreting our findings it is important to consider the breadth of the food groups we describe; broader food groups are more likely to appear as top contributors to nutrient intakes and vice versa. Our approach was to create more food groups than will be necessary to answer most research questions, leaving it open to researchers to collapse categories to create larger groupings. For example, meats are reported as beef, pork, lamb etc. rather than “red meat”, so that a reader interested in the contribution of total red meat to saturated fat would need to add the components together.

Overall the top food groups contributing to energy intake in this sample of British adults were consistent with the more disaggregated food groups reported by the UK NDNS [17]: desserts, cakes and pastries; white bread; white pasta/rice; fruit; semi-skimmed milk; biscuits; fried/roast potatoes; wholemeal/mixed bread and high-fat cheese. Some of these foods are high in saturated fat and free sugars, contributing to excess intakes of these nutrients relative to dietary guidelines which are associated with ill-health, including obesity, diabetes and cardiovascular disease risk [18–23]. Some food groups (e.g. desserts) which are top contributors to energy, SFA, and free sugars are also important sources of calcium and iron (partly as a result of fortification), which will need to be replaced by other food sources of these important micronutrients if the overall diet quality is to be enhanced. Dairy products such as semi-skimmed milk and high fat cheese were the major contributors to total energy intakes as well as total fat, saturated fat, total protein, vitamin B12 and calcium. Most of the dietary fibre was obtained from wholemeal and mixed/granary breads, however white bread does not contribute much to the fibre intakes but was highly consumed (providing 3.4% EI overall compared to 2.7%EI of wholemeal and mixed/granary). This illustrates the potential for swapping refined grains with whole grains to make substantial progress towards dietary fibre recommendations which are associated with improved health outcomes [24, 25].

Our analyses also highlight the large contribution of alcoholic drinks to total energy intake in this population, averaging approximately 7% EI from all alcoholic beverages, greatly exceeding the contribution of fruit juice (1.8% EI) or sugar-sweetened beverages (1.4% EI). In addition to increasing the risk of excess weight gain as a consequence of increased energy intake, this raises concerns about alcohol-related harms. Although meta-analyses of observational studies have found positive associations of moderate alcohol intake for the prevention of coronary heart disease [26], other studies have also found a higher risk of different types of stroke and other CVD subtypes [27], and several types of cancer [28], which support current guidance to limit consumption of alcoholic beverages. Among non-alcoholic beverages, tea and water contributed the most to the total daily beverage intake (gr). However, fruit juice and sugar-sweetened beverages were top sources of total energy intake from non-alcoholic beverages and contributed the most to free sugar intakes, which have been linked to increased risk of weight gain and increased cardiometabolic risk [18, 29–32].

We also found relatively few differences in intakes between people with different levels of education, which may reflect the limited variation in educational attainment in this cohort as well as a “healthy volunteer” bias related to the selection of participants who completed the dietary questionnaires [6]. The major differences found here were in beverage consumption and, as a result, people with lower levels of education consumed proportionally more free sugars, in the form of SSBs and beer/cider, whereas among people with higher levels of education fruit juice is the major contributor, which has previously been reported in NDNS [17]. There were also differences in the sources of saturated fat, with high-fat cheese being proportionally more important among people with higher education, compared to biscuits and chocolate confectionery in lower education groups. This is consistent with an analysis of purchases from 25,674 British households showing that dairy sources (mostly cheese) contributed more to SFA in higher socioeconomic groups [4]. These differences are small but may still reflect variability in dietary quality and contribute to inequalities in diet and health across SES groups which have been widely documented in the literature [4, 33, 34].

In conclusion, this work has developed a food grouping system which will be available to all studies using the Oxford WebQ, including the whole UK Biobank community. We encourage researchers to make use of these food groups in future studies to generate more consistent evidence which can inform food-based dietary guidelines or advice for the public to reduce health risks.

## Supplementary Information

Below is the link to the electronic supplementary material.

Supplementary file1 (DOCX 30 kb)

## Data Availability

Researchers can apply to use the UK Biobank resource and access the data used. Code for this analysis can be available upon request to the investigators.
