# Correction to: Describing a new food group classification system for UK biobank: analysis of food groups and sources of macro- and micronutrients in 208,200 participants

**DOI:** 10.1007/s00394-021-02591-3

**Published:** 2021-06-10

**Authors:** Carmen Piernas, Aurora Perez-Cornago, Min Gao, Heather Young, Zoe Pollard, Angela Mulligan, Marleen Lentjes, Jennifer Carter, Kathryn Bradbury, Tim J. Key, Susan A. Jebb

**Affiliations:** 1 Nuffield Department of Primary Care Health Sciences, University of Oxford, Radcliffe Primary Care Building, Radcliffe Observatory Quarter, Woodstock Road, Oxford, OX2 6GG UK; 2 Cancer Epidemiology Unit, Department of Population Health, University of Oxford, Nuffield, UK; 3 School of Public Health, Peking University, Beijing, China; 4 Department of Public Health & Primary Care, Institute of Public Health, University of Cambridge, Cambridge, UK; 5 NIHR BRC Diet, Anthropometry and Physical Activity Group, MRC Epidemiology Unit, University of Cambridge, Cambridge, UK; 6 School of Medical Sciences, Clinical Epidemiology & Biostatistics, Örebro University, Örebro, Sweden; 7 Nuffield Department of Population Health, University of Oxford, Oxford, UK; 8 School of Population Health, National Institute for Health Innovation, University of Auckland, Auckland, New Zealand

## Correction to: European Journal of Nutrition 10.1007/s00394-021-02535-x

The original version of this article unfortunately contained a mistake. In figure 1, panel for Fibre (top right hand side) was wrong.

The corrected Fig. 1 is given in the following page.

**Fig. 1:**
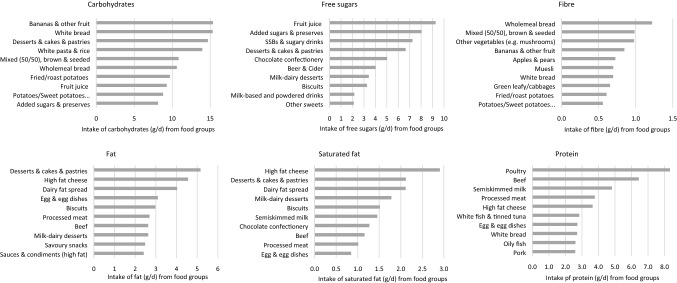
Top 10 food contributors to total carbohydrates, free sugars, fibre, total fat, saturated fat and total protein per capita among all UK Biobank participants

Reference 11 should read:

Perez-Cornago A, Pollard Z, Young H, van Uden M, Andrews C, Piernas C, Key TJ, Mulligan A, Lentjes M (2021) Description of the updated nutrition calculation of the Oxford WebQ questionnaire and comparison with the previous version among 207,144 participants in UK Biobank. Eur J Nutr. https://doi.org/10.1007/s00394-021-02558-4

The original article has been corrected.

