# Supplementary material for: Describing a new food group classification system for UK biobank: analysis of food groups and sources of macro- and micronutrients in 208,200 participants
Source: Eur J Nutr. 2021 Mar 25;60(5):2879–90. doi: 10.1007/s00394-021-02535-x (PMC8275520; doi:10.1007/s00394-021-02535-x)
Supplement: Supplementary file 1 — Supplementary file1 (DOCX 30 kb) [file 394_2021_2535_MOESM1_ESM.docx]

# Online Supplemental Material

Supplementary Table 1. Food group system

| **Name** | **Includes** |
| --- | --- |
| ***1) Cereals & cereal products*** |  |
| **White bread** | White bread, sliced, baguette, bap, roll |
| **Wholemeal bread** | Wholemeal bread, sliced, baguette, bap, roll |
| **Mixed bread, brown & seeded** | Mixed, brown or seeded bread, sliced, baguette, bap, roll |
| **Other bread** | Naan, garlic bread, other bread (including gluten free) |
| **Savoury crackers** | Oatcakes, crispbreads (including gluten free) |
| **Bran cereal** | Bran cereal |
| **Biscuit cereal** | Wholewheat biscuit cereal |
| **Oat cereal (non-sugar)** | Porridge oats (including milk/dried fruit added) |
| **Oat cereal (sugar)** | Oatcrunch breakfast cereal |
| **Muesli** | Muesli (with or without dried fruit) |
| **Other cereal (sugar)** | Plain and sugary breakfast cereal (with/without dried fruit) |
| **White pasta & rice** | White pasta, rice, couscous, gluten free pasta |
| **Wholemeal pasta, brown rice & other wholegrains** | Brown and wholemeal pasta and rice |
| ***2) Mixed-dishes*** |  |
| **Pizza** | Pizza (including gluten free crust) |
| **Grain dishes - added fat** | Double and single crust pies, crumble pies, Yorkshire pudding, snackpot noodles |
| **Samosa, pakora** | Indian samosa, pakora snacks |
| **Soups** | Soups, homemade, powdered and canned |
| **Sushi** | Sushi |
| ***3) Dairy & dairy-free products*** |  |
| **Whole milk** | Whole milk >3.6 g fat per 100 g (cow, goat, sheep) |
| **Semi-skimmed milk** | Semi-skimmed milk >1 g fat per 100 g (cow, other) |
| **Skimmed milk** | Skimmed milk <1 g fat per 100 g (cow, cholesterol lowering, powdered) |
| **Rice/oat drink** | Rice and oat vegetable drinks |
| **Soy drink** | Soya drinks (including calcium fortified) |
| **Full fat yogurt** | Whole milk yogurt (plain) |
| **Low fat yogurt** | Fat free and lower fat yogurt, plain or flavoured |
| **High fat cheese** | Cheese >17.5 g fat per 100 g, including hard cheese, soft cheese, spreadable, Blue, Feta, Mozzarella, Goats, other) |
| **Medium & low fat cheese** | Cheese <=17.5g fat per 100 g, including hard and spreadable lower fat cheese, Cottage |
| **Cream** | Cream (cow’s milk) |
| ***4) Egg & egg dishes*** |  |
| ***Egg and egg dishes*** | Whole eggs and processed (omelette, scotch eggs, other) |
| ***5) Fat & spreads*** |  |
| **Olive oil (drizzling/dunking)** | Olive oil |
| **Dairy fat spread lower fat** | Spreadable/lower fat butter, dairy-based very low fat spread |
| **Dairy fat spread** | Spreadable normal fat butter, dairy-based normal fat spread (including cholesterol lowering spread) |
| **Vegetable spread lower fat** | Olive oil based lower fat spread, plant-based lower fat margarine and soya-based lower fat spread (including cholesterol lowering spread) |
| **Vegetable spread** | Olive oil based spread, plant-based soft or hard margarine and soya-based spread (including cholesterol lowering spread) |
| ***6) Meat & meat products*** |  |
| **Poultry** | Poultry (with/without skin) |
| **Pork** | Pork |
| **Beef** | Beef |
| **Lamb** | Lamb |
| **Other meat, offal** | Other meat including offal |
| **Processed meat** | Sausages, bacon (with and without fat), ham, liver pate |
| **Breaded/battered Chicken** | Fried poultry with batter/breadcrumbs |
| ***7) Fish & fish dishes*** |  |
| **White fish & tinned tuna** | Tinned tuna, white fish, other fish |
| **Shellfish** | Prawns, lobster, crab, shellfish |
| **Oily fish** | Oily fish, including salmon, |
| **Breaded/battered Fish** | Fried fish with batter/breadcrumbs |
| ***8) Meat substitutes*** |  |
| **Vegetarian meals** | Quorn-based and vegetarian burgers and products |
| **Soy-based meals** | Tofu-based products |
| ***9) Vegetables & potatoes*** |  |
| **Raw salad** | Mixed side salad, lettuce, watercress |
| **Green leafy/cabbages** | Broccoli, cabbage, kale, cauliflower, spinach, sprouts |
| **Root vegetables** | Beetroot, carrots, celery, parsnip, turnip |
| **Tomatoes** | Fresh and tinned tomatoes |
| **Allium vegetables** | Garlic, leek, onion |
| **Other vegetables (mushrooms, fruiting, mixed)** | Mushrooms, mixed vegetables, avocado, broad beans, green beans, butternut squash, courgettes, , peppers, other |
| **Peas/sweetcorn** | Peas, sweetcorn |
| **Potatoes/sweet potatoes (baked/boiled)** | Potatoes, sweet potatoes, boiled or baked |
| **Mashed potatoes** | Potatoes, mashed |
| **Fried/roast potatoes** | Potatoes and chips, fried or roasted with fat |
| **Legumes/pulses** | Baked beans, pulses |
| **Vegetable side dishes** | Coleslaw, salad with added fat/mayonnaise |
| **Vegetable dips** | Hummus, guacamole |
| ***10) Fruits*** |  |
| **Citrus** | Grapefruit, orange, satsuma |
| **Berries** | Blackberries, strawberries, blueberries, raspberries, cherries |
| **Apples & pears** | Apples and pears |
| **Bananas & other fruit** | Bananas, mixed fruit, grapes, mango, melon, peach, pineapple, kiwi, other |
| **Dried fruit** | Dried fruit, prunes |
| **Stewed fruit** | Stewed fruit, plums |
| ***11) Nuts & seeds*** |  |
| **Salted nuts & seeds** | Salted peanuts and nuts |
| **Unsalted nuts & seeds** | Unsalted peanuts and nuts |
| ***12) Sugar, preserves, cakes & confectionery, snacks*** |  |
| **Added sugars & preserves** | Table sugar, honey, jam and preserves |
| **Chocolate confectionery** | Chocolate bar (including white, milk and dark chocolate), chocolate-covered raisins, chocolate-covered sweets |
| **Other sweets** | Hard and soft sweets (including sugar free) |
| **Savoury snacks** | Crisps, savoury biscuits, cheese snacks, other savoury biscuits |
| **Biscuits** | Chocolate biscuits, plain biscuits, sweet biscuits and cookies |
| **Milk-dairy desserts** | Ice cream, milk puddings, milk-based desserts, cheesecake |
| **Desserts & cakes & pastries** | Pancakes, croissant, Danish pastries, scones, fruitcakes, cakes, doughnuts, sponge puddings, other desserts, cereal bars, sweet snacks |
| **Soya-based desserts & yogurt** | Soya-based desserts |
| **Nut-based spreads** | Peanut-butter and chocolate-based spread |
| ***13) Sauces & condiments*** |  |
| **Sauces (higher fat)** | Mayonnaise, salad dressing, pesto, cheese sauce, white sauce, gravy |
| **Sauces (lower fat)** | Yeast, chutney, olives, ketchup, brown sauce, tomato sauce |
| ***14) Non-alcoholic beverages**** |  |
| **Fruit juice** | Orange, grapefruit drink and 100% fruit juice |
| **Coffee, caffeinated** | Normal instant, filter, cappuccino, espresso coffee |
| **Coffee, decaffeinated** | Decaffeinated instant, filter, cappuccino, espresso coffee |
| **Tea** | Black, green and other tea |
| **Tea, decaffeinated** | Decaffeinated black, herbal tea, rooibos |
| **SSBs & other sugary drinks** | Fizzy sugary drinks, squash, fruit smoothies |
| **Low/non sugar SSBs** | Low calorie fizzy drinks and squash |
| **Water/sparkling water** | Plain water, sparkling water |
| **Milk-based & powdered drinks** | Dairy-based smoothies, milk-based drinks, hot chocolate |
| ***15) Alcoholic beverages**** |  |
| **White wine** | White wine |
| **Red wine** | Red and rose wine |
| **Fortified wine** | Fortified wine |
| **Beer & Cider** | Beer and cider |
| **Spirits** | Spirits and other alcoholic drinks |
| *Units for beverages are ml/day |  |

Supplemental Table 2. Demographic characteristics of the study population

|  | **Total** | **Women** | **Men** |
| --- | --- | --- | --- |
|  | **n=208200** | **n=114965** | **n=93235** |
| **Age at recruitment, mean (SD)** | 56.1 (7.9) | 55.7 (7.8) | 56.7 (8.0) |
| **% White ethnicity** | 198959 (95.6%) | 109797 (95.5%) | 89162 (95.6%) |
| **% Higher education degree** | 100336 (48.2%) | 55888 (48.6%) | 44448 (47.7%) |
| **% High physical activity** | 74892 (36.0%) | 39145 (34.0%) | 35747 (38.3%) |
| **% Current smoker** | 16226 (7.8%) | 7646 (6.7%) | 8580 (9.2%) |
| **Alcohol** |  |  |  |
| **5 or more units/week** | 47838 (23.0%) | 21507 (18.7%) | 26331 (28.2%) |
| **1-4 units/week** | 103879 (49.9%) | 55513 (48.3%) | 48366 (51.9%) |
| **<1 units /week** | 43363 (20.8%) | 29502 (25.7%) | 13861 (14.9%) |
| **Never** | 12966 (6.2%) | 8353 (7.3%) | 4613 (4.9%) |
| **Body mass index (BMI), mean (SD)** | 26.9 (4.6) | 26.5 (5.0) | 27.4 (4.1) |
| **BMI group** |  |  |  |
| **Underweight (<18.5)** | 1136 (0.5%) | 941 (0.8%) | 195 (0.2%) |
| **Normal (18.5 to <25)** | 76949 (37.0%) | 50518 (43.9%) | 26431 (28.3%) |
| **Overweight (25 to <30)** | 86487 (41.5%) | 40417 (35.2%) | 46070 (49.4%) |
| **Obesity (≥30)** | 43535 (20.9%) | 23059 (20.1%) | 20476 (22.0%) |
| * Descriptive statistics: n(%) or means (SD) | | | |

Supplemental Table 3. Top ten food groups contributing to daily intake per capita (g/day) of carbohydrates, free sugars, fibre, total protein, total fat and saturated fat among all UK Biobank participants

|  | **Mean SD (g/d)** |  | **Mean SD (g/d)** |  | **Mean SD (g/d)** |
| --- | --- | --- | --- | --- | --- |
| **Total daily carbohydrate** | 255.6 (78) | **Total daily free sugars** | 61.1 (36.2) | **Total daily Englyst fibre** | 17.9 (6.6) |
| ***Carbohydrate intake from:*** |  | ***Free sugar intake from:*** |  | ***Fibre intake from:*** |  |
| Bananas & other fruit | 15.4 (14.9) | Fruit juice | 9.3 (12.7) | Wholemeal bread | 1.2 (2.0) |
| White bread | 15.3 (24.3) | Added sugars & preserves | 8.0 (13.6) | Mixed (50/50), brown & seeded | 1.0 (1.6) |
| Desserts & cakes & pastries | 14.7 (18.8) | SSBs & other sugary drinks | 7.2 (14.9) | Other vegetables(e.g. mushrooms, etc) | 1.0 (1.2) |
| White pasta & rice | 14.0 (22.5) | Desserts & cakes & pastries | 6.6 (9.0) | Bananas & other fruit | 0.8 (0.9) |
| Mixed bread, brown & seeded | 10.8 (17.7) | Chocolate confectionery | 5.0 (10.4) | Apples & pears | 0.7 (1.0) |
| Wholemeal bread | 10.6 (17.5) | Beer & Cider | 4.0 (10.7) | Muesli | 0.7 (1.5) |
| Fried/roast potatoes | 9.7 (19.0) | Milk-dairy desserts | 3.4 (6.6) | White bread | 0.7 (1.1) |
| Fruit juice | 9.3 (12.7) | Biscuits | 3.3 (5.1) | Green leafy/cabbages | 0.7 (1.1) |
| Potatoes/Sweet potatoes (baked/boiled) | 8.8 (13.6) | Milk-based & powdered drinks | 2.2 (5.8) | Fried/roast potatoes | 0.6 (1.2) |
| Added sugars & preserves | 8.1 (13.7) | Other sweets | 2.1 (9.9) | Potatoes/Sweet potatoes (baked/boiled) | 0.6 (0.9) |
|  |  |  |  |  |  |
|  | **Mean SD (g/d)** |  | **Mean SD (g/d)** |  | **Mean SD (g/d)** |
| **Total daily fat** | 73.5 (28.3) | **Total daily saturated fat** | 27.2 (11.9) | **Total daily protein** | 81.1 (24.3) |
| ***Fat intake from:*** |  | ***Saturated fat intake from:*** |  | ***Protein intake from:*** |  |
| Desserts & cakes & pastries | 5.2 (7.4) | High fat cheese | 2.9 (3.9) | Poultry | 8.3 (13.5) |
| High fat cheese | 4.6 (6.1) | Desserts & cakes & pastries | 2.1 (3.1) | Beef | 6.4 (11.7) |
| Dairy fat spread | 4 (7.3) | Dairy fat spread | 2.1 (4.0) | Semi-skimmed milk | 4.8 (4.8) |
| Egg & egg dishes | 3.1 (6.1) | Milk-dairy desserts | 1.8 (3.6) | Processed meat | 3.8 (6.5) |
| Biscuits | 3 (4.5) | Biscuits | 1.5 (2.3) | High fat cheese | 3.6 (4.9) |
| Processed meat | 2.7 (5.3) | Semi-skimmed milk | 1.5 (1.4) | White fish & tinned tuna | 2.8 (6.9) |
| Beef | 2.6 (5.0) | Chocolate confectionery | 1.3 (2.7) | Egg & egg dishes | 2.7 (5.0) |
| Milk-dairy desserts | 2.6 (5.5) | Beef | 1.2 (2.2) | White bread | 2.7 (4.3) |
| Savoury snacks | 2.5 (4.5) | Processed meat | 1.0 (2.0) | Oily fish | 2.6 (6.3) |
| Sauces & condiments (high fat) | 2.4 (4.0) | Egg & egg dishes | 0.8 (1.8) | Pork | 2.6 (8.2) |

Supplemental Table 4. Top ten food groups contributing to daily intake per capita of folate, B12, vitamin C, iron, calcium and potassium among all UK Biobank participants

|  | **Mean SD**  **(µg/d)** |  | **Mean SD**  **(µg/d)** |  | **Mean SD**  **(mg/d)** |
| --- | --- | --- | --- | --- | --- |
| **Total daily folate** | 313.57 (107.23) | **Total daily B12** | 6.18 (3.32) | **Total daily vitamin C** | 128.44 (77.98) |
| ***Folate intake from:*** |  | ***B12 intake from:*** |  | ***Vitamin C intake from:*** | |
| Other vegetables (e.g.mushrooms) | 20.71 (25.50) | Semi-skimmed milk | 1.12 (1.11) | Fruit juice | 31.24 (43.88) |
| Fruit juice | 18.06 (27.77) | Oily fish | 0.77 (1.87) | Other vegetables (e.g.mushrooms) | 15.55 (28.61) |
| Green leafy/cabbages | 14.77 (25.93) | Beef | 0.58 (1.05) | Citrus fruit | 14.79 (25.37) |
| Mixed bread, brown & seeded | 12.05 (19.95) | Processed meat | 0.44 (1.88) | Bananas & other fruit | 12.05 (16.72) |
| Tea | 11.79 (10.38) | Egg & egg dishes | 0.44 (0.81) | Green leafy/cabbages | 11.39 (19.67) |
| Semi-skimmed milk | 11.22 (11.10) | Skimmed milk | 0.39 (0.86) | Tomatoes | 7.05 (9.85) |
| Bananas & other fruit | 10.63 (11.34) | White fish & tinned tuna | 0.36 (0.92) | SSBs & other sugary drinks | 4.90 (11.52) |
| Bran cereal | 10.54 (30.62) | High fat cheese | 0.25 (0.34) | Potatoes/Sweet potatoes (baked/boiled) | 3.95 (6.21) |
| Wholemeal bread | 10.43 (17.16) | Lamb | 0.17 (0.61) | Berries | 2.85 (6.14) |
| Other cereal (sugar) | 10.04 (23.31) | Oat cereal (non-sugar) | 0.17 (0.40) | Semi-skimmed milk | 2.79 (2.77) |
|  |  |  |  |  |  |
|  | **Mean SD (mg/d)** |  | **Mean SD (mg/d)** |  | **Mean SD**  **(mg/d)** |
| **Total daily iron** | 12.4 (3.79) | **Total daily calcium** | 988.62 (340.67) | **Total daily potassium** | 3681.63 (1058.65) |
| ***Iron intake from:*** |  | ***Calcium intake from:*** |  | ***Potassium intake from:*** | |
| Wholemeal bread | 0.60 (0.99) | Semi-skimmed milk | 167.46 (165.68) | Bananas & other fruit | 241.8 (233.39) |
| Red wine | 0.59 (1.18) | High fat cheese | 93.95 (129.45) | Semi-skimmed milk | 216.98 (215.03) |
| Beef | 0.54 (0.99) | Skimmed milk | 57.78 (127.56) | Fried/roast potatoes | 192.41 (378.04) |
| White bread | 0.52 (0.82) | Low fat yogurt | 50.97 (81.19) | Potatoes/Sweet potatoes (baked/boiled) | 190.05 (295.23) |
| Other cereal (sugar) | 0.47 (1.06) | White bread | 47.08 (75.04) | Fruit juice | 143.67 (196.48) |
| Mixed bread, brown & seeded | 0.47 (0.76) | Mixed bread, brown & seeded | 40.06 (65.58) | Coffee, caffeinated | 124.57 (145.39) |
| Desserts & cakes & pastries | 0.46 (0.63) | Oat cereal (non-sugar) | 38.94 (85.00) | Other vegetables (e.g. mushrooms) | 117.86 (146.12) |
| Biscuit cereal | 0.46 (1.13) | Milk-based & powdered drinks | 28.49 (80.11) | Tea | 109.29 (91.22) |
| Bran cereal | 0.43 (1.24) | Wholemeal bread | 25.53 (41.77) | Poultry | 100.32 (162.55) |
| Egg & egg dishes | 0.37 (0.69) | Desserts & cakes & pastries | 24.42 (34.50) | Tomatoes | 92.88 (129.59) |
